# Antibacterial 3D-Printed Silver Nanoparticle/Poly Lactic-Co-Glycolic Acid (PLGA) Scaffolds for Bone Tissue Engineering

**DOI:** 10.3390/ma16113895

**Published:** 2023-05-23

**Authors:** Fajun Chen, Jian Han, Zeyong Guo, Chongjing Mu, Chuandi Yu, Zhibo Ji, Lei Sun, Yujuan Wang, Junfeng Wang

**Affiliations:** 1 Department of Anatomy, School of Basic Medicine, Anhui Medical University, No.81, Meishan Road, Shushan District, Hefei 230032, China; 2 High Magnetic Field Laboratory, Hefei Institutes of Physical Science, Chinese Academy of Sciences, Science Island, Hefei 230031, China; 3 Graduate School of University of Science and Technology of China, Hefei 230026, China; 4 The Affiliated Suzhou Hospital of Nanjing Medical University, 16 Baita West Road, Suzhou 215000, China; 5 Department of Stomatology, The Second Affiliated Hospital of Anhui Medical University, Hefei 230601, China; 6 Department of Oral Surgery, Ninth People’s Hospital, College of Stomatology, Shanghai Jiao Tong University School of Medicine, Shanghai Key Laboratory of Stomatology & Shanghai Research Institute of Stomatology, National Clinical Research Center of Stomatology, Shanghai 200011, China

**Keywords:** PLGA, AgNPs, bone tissue engineering, antibacterial scaffold, 3D printing

## Abstract

Infectious bone defects present a major challenge in the clinical setting currently. In order to address this issue, it is imperative to explore the development of bone tissue engineering scaffolds that are equipped with both antibacterial and bone regenerative capabilities. In this study, we fabricated antibacterial scaffolds using a silver nanoparticle/poly lactic-co-glycolic acid (AgNP/PLGA) material via a direct ink writing (DIW) 3D printing technique. The scaffolds’ microstructure, mechanical properties, and biological attributes were rigorously assessed to determine their fitness for repairing bone defects. The surface pores of the AgNPs/PLGA scaffolds were uniform, and the AgNPs were evenly distributed within the scaffolds, as confirmed via scanning electron microscopy (SEM). Tensile testing confirmed that the addition of AgNPs enhanced the mechanical strength of the scaffolds. The release curves of the silver ions confirmed that the AgNPs/PLGA scaffolds released them continuously after an initial burst. The growth of hydroxyapatite (HAP) was characterized via SEM and X-ray diffraction (XRD). The results showed that HAP was deposited on the scaffolds, and also confirmed that the scaffolds had mixed with the AgNPs. All scaffolds containing AgNPs exhibited antibacterial properties against *Staphylococcus aureus* (*S. aureus*) and *Escherichia coli* (*E. coli*). A cytotoxicity assay using mouse embryo osteoblast precursor cells (MC3T3-E1) showed that the scaffolds had excellent biocompatibility and could be used for repairing bone tissue. The study shows that the AgNPs/PLGA scaffolds have exceptional mechanical properties and biocompatibility, effectively inhibiting the growth of *S. aureus* and *E. coli*. These results demonstrate the potential application of 3D-printed AgNPs/PLGA scaffolds in bone tissue engineering.

## 1. Introduction

In recent years, the repair of bone defects has represented a great challenge and research hotspot in the global medical field, and a large number of patients require the reconstruction of bone defects every year due to congenital diseases, trauma, infection, and tumors [1]. At the same time, large bone defects are frequently associated with a high risk of infection, which is one of the most important clinical factors leading to bone implant failure [2]. Pathogenic bacteria can significantly damage local tissues and any bone regeneration capacity, resulting in local blood damage and osteonecrosis. In order to address this problem, the common clinical treatment used is antibiotics, but it is difficult to achieve effective drug concentrations at the infected site through the systemic administration of antibiotics, which may lead to a delay in bone healing, non-healing, amputation, or sometimes even death [3,4,5]. Thus, in order to reduce the incidence of infection and ensure a good prognosis, the development of a bifunctional biologic bone scaffold with antibacterial properties and a bone defect reconstruction capacity is essential [6].

Charles Hull reported 3D printing technology for the first time in 1986 [7]. With the development of 3D printing technology in the fields of biomedicine and tissue engineering, it is now one of the most promising methods for manufacturing bone scaffolds. Using computer-aided design (CAD), 3D printing technology permits the fabrication of scaffolds with precise geometries [6]. When compared to traditional manufacturing techniques, 3D printing technology is increasingly used in the fields of tissue engineering and medical regeneration due to its characteristics, such as rapid prototyping and personalization [8]. In addition, 3D printing allows for the precise control of pore connectivity, porosity, and pore-space distribution in scaffolds [8], which are crucial factors in the applications of bone tissue engineering scaffolds for cell permeation, fluid flow, and metabolite removal [9]. Direct ink writing (DIW) is a simple and cost-effective technique for 3D printing technologies, and it is also the most popular 3D printing method [10]. The key characteristic valued in DIW is the ability to customize the inks freely and to print 3D structures at mesoscales and microscales precisely [11]. The richness in the potential of DIW has led to extensive research [12]. Many biological materials can be printed using DIW, including polymers, ceramics, glass, cement, graphene, metals, and their combinations [10]. Guo et al. used the DIW fabrication method, which allows 3D geometries to be created at room temperature by mixing the dissolvable polymer polylactic acid with dichloromethane, in a free-form fashion [13]. At the same time, some researchers have successfully fabricated bone scaffolds using ceramic-based bioactive materials via DIW printing technology. For example, Sun et al. successfully fabricated bone scaffolds for skull defect repair by mixing Wollastonite-Mg powders with a polyvinyl alcohol solution via the DIW technique [14]. In addition, another important feature of DIW is its ability to mix materials together evenly; for example, carbon-based nanomaterials, such as graphene, can be uniformly added to the polymer ink to print multifunctional 3D composites [10].

Polylactic-co-glycolic acid (PLGA) is a polymeric organic compound synthesized via the polymerization of two monomers: lactic acid and glycolic acid. It has good biocompatibility, adjustable degradability and mechanical properties, and shows no biological toxicity [15]. This is why it is widely used in bone tissue engineering. PLGA is degraded in the human body to lactic acid and glycolic acid, both of which are body metabolites and can be eliminated naturally [16]. In order to conform to the processing mode of 3D printing, organic solvents, such as acetone and 1,4-dioxane, are typically employed to dissolve PLGA in order to prepare the printing inks [8]. Antibiotics are typically utilized to impart antimicrobial properties to scaffolds. Visscher et al. produced 3D-printed polycaprolactone (PCL) scaffolds with macro- and micropores and an antibiotic coating (cefazolin). The scaffolds showed antimicrobial activity against *S. aureus* [17]. Another group printed PCL/PLGA scaffolds loaded with tobramycin and demonstrated antibacterial activity against *S. aureus* in vitro, treating the established bone infection in a rat model successfully [18,19]. Despite these promising designs, the growing concern about antibiotic-resistant bacteria highlights the need for developing alternative methods [20].

Silver nanoparticles (AgNPs) have attracted considerable attention due to their low level of cytotoxicity, remarkable antibacterial properties, and comparatively low bacterial resistance [21,22]. They can be blended directly into inorganic materials or combined with synthetic biopolymer materials to produce novel antibacterial materials. Studies have shown that AgNPs hold strong antibacterial, antiviral, and anti-eukaryotic properties [23]. The antimicrobial effect of AgNPs has been widely studied, and the mechanisms are being explored. The antibacterial potency of AgNPs is largely attributed to the oxidative form of silver ions, which bind to and penetrate bacterial cell walls, resulting in the disruption of cell membranes [24]. It can also interact with the thiol groups of key enzymes and effectively inactivate them. Furthermore, silver ions target the sulfur and phosphorus elements in DNA, inhibiting DNA replication [25]. Additionally, AgNPs lead to the collapse of the membrane proton gradient and the destruction of many mechanisms of cell metabolism, resulting in cell death [26]. AgNPs are widely used in commercial biomedical products, such as catheters and wound dressings, due to their strong antibacterial properties [21].

In order to address the problems encountered in repairing bone defects, we combined biocompatible and biodegradable PLGA with strongly antibacterial AgNPs. A promising AgNPs/PLGA bone scaffold was synthesized using DIW, which can precisely control the scaffold’s aperture, pore connectivity, porosity, and pore spatial distribution. Unlike traditional surface coating [27] and soaking [28], the AgNPs incorporated via the DIW are evenly distributed in the scaffolds and slowly released in the body, thereby eliminating the risk of bacterial infection during the later stages of bone grafting by consistent and stable antimicrobial effect.

In this study, we blended AgNPs with PLGA in a specific proportion and dissolved them in trichloromethane. By using DIW 3D printing technology, we fabricated bone scaffolds with AgNPs evenly distributed in the PLGA material. We then assessed the scaffolds’ antibacterial efficacy in vitro and evaluated their biocompatibility using mouse embryo osteoblast precursor cells (MC3T3-E1) to determine their potential for use in bone tissue engineering.

## 2. Materials and Methods

### 2.1. Characterization of the AgNPs Scaffolds

The particle size of AgNPs (Zhejiang Yami Nano Technology Co., Jiaxing, China) was measured by using dynamic light scattering (DLS, Dynapro Titan TC, Santa Barbara, CA, USA). The microscopic morphology of AgNPs was observed by using a ZESS ΣIGMA field emission scanning electron microscope (SEM).

### 2.2. Preparation of the AgNPs/PLGA Composites

A total of 0.5 g of AgNPs were weighed into a 10 mL centrifuge tube and mixed with 5 mL trichloromethane via ultrasonication. AgNPs with 3 different percentages (3%, 1%, and 0.1% wt%) were added into the PLGA, respectively, as follows: 3AgNPs/PLGA (3% AgNPs), 1AgNPs/PLGA (1% AgNPs), and 0.1AgNPs/PLGA (0.1% AgNPs), respectively. According to the concentration requirements, 2 g PLGA (DG-85DLG300) was mixed with the AgNPs solution (0.6 mL, 0.2 mL, and 0.02 mL), and trichloromethane was added up to 3 mL, and then an agitator (ARV-310, Thinky, Kyoto, Japan) was used to mix for 15 min in order to dissolve the PLGA completely as well as ensuring the PLGA and AgNPs were uniformly mixed.

### 2.3. Preparation of 3D-Printed AgNPs/PLGA Scaffolds

DIW was utilized to fabricate AgNPs/PLGA scaffolds. Solidwork 2022 software was used to develop the scaffold models. Subsequently, printing was accomplished utilizing an DIW-based 3D printer (Ultimaker 2+ Extended, Ultimaker, Utrecht, The Netherland); the printing nozzle model was a MUSASHI TPND-25G (the size of the printing nozzle was 0.25 mm), while the printing syringe model was a MUSASHI PSY-50E. During the fabrication process, the AgNPs/PLGA mixture was extruded at 250 kPa air pressure and deposited onto the print bed by forming filaments through the nozzles, moving the printing head along the *X* and *Y* axes, and lowering the printing bed in the *Z*-axis direction for layer-by-layer deposition to construct a complete 3D structure. After each layer was deposited, the printing bed was lowered, and the subsequent layer was deposited on the top of the previously deposited layer. The following 3D printing parameters were set: printing speed: 35%, nozzle temperature: 3 °C; platform temperature: 3 °C; the length, width, and height of the scaffolds were 1 mm, 2 cm, and 2 cm, respectively.

### 2.4. Characterization of the AgNPs/PLGA Scaffolds

#### 2.4.1. Microstructure of the Scaffolds

The microstructure and surface morphology of the samples were observed via SEM with a scanning voltage of 5 kV. Prior to testing, the scaffolds (both sides and cross sections) were sputter-coated with gold particles to increase their electrical conductivity.

#### 2.4.2. Mechanical Properties of the Scaffolds

Mechanical property tests of the scaffolds were measured using an AGS-X electromechanical test frame (SHIMADZU, Kyoto, Japan) with 10 mm/min displacement rate, according to the American Society for Testing and Materials (ASTM) D638 standard. The Young’s modulus and stress-strain curves of the AgNPs/PLGA scaffolds were obtained from the tensile test to evaluate their mechanical properties. The size of the scaffolds used for the mechanical property tests was 6 cm × 1 cm × 0.1 cm.

#### 2.4.3. The Release of Silver Ions from the Scaffolds In Vitro

AgNPs/PLGA scaffolds (0.1 g), with various mass ratios, were placed in a 50 mL centrifuge tube, and 20 mL of deionized water was added. The tubes were gently shaken until the scaffolds were fully submerged in water, following which they were incubated at 37 °C. Subsequently, 3.5 mL of the deionized water samples were collected on days 7, 14, 21, and 28 and filtered with a 0.22 μm filter tip. The concentration of silver ions in the solution was then measured by using inductively coupled plasma atomic emission spectrometry (ICP-7400, Thermo Fisher, Waltham, MA, USA). In addition, the scaffolds were immersed in deionized water, and their colorimetric change was observed to determine if silver ions were released from the scaffolds.

#### 2.4.4. Chemical Characterization of the Scaffolds

A qualitative evaluation of the composition of the scaffolds was performed via Fourier transform infrared spectroscopy (FTIR, Invenio-R, Bruker, Billerica, MA, USA).

#### 2.4.5. Biological Characterization of the Scaffolds

##### X-ray Diffraction (XRD) Characterization of the Scaffolds

A bioactivity test was performed on triplicate samples using an in vitro method by immersing them in simulated body fluid (SBF) [29]. The samples were incubated in SBF at 37 °C for 14 days and then dried overnight in an oven and examined under SEM to observe the growth of the hydroxyapatite (HAP) layer on their surfaces. The HAP layers that grew were then analyzed further via XRD to confirm their identity.

##### Cell Proliferation

The 5 mm diameter scaffolds were soaked in ethanol for 2 h and then washed 3 times with sterile PBS and finally exposed to UV light overnight. Subsequently, cytotoxicity experiments were performed using MC3T3-E1 (Chinese Academy of Medical Sciences, Beijing, China). A T25 cell culture dish was selected, and Dulbecco’s Modified Eagle’s Medium (DMEM, Corning) containing 10% fetal bovine serum (FBS, Biochrom AG, Berlin, Germany) and 5% penicillin/streptomycin (P/S, Biochrom AG) were added to the culture medium. The cells were revived and incubated at 37 °C and 5% CO_2_ in an incubator with a constant temperature. After the cells had colonized the entire culture dish, they were digested with trypsin (Gibco, NY, USA), and the number of the cells was determined. Each well of the 96-well plate was seeded with 1 × 10^3^ cells. Each scaffold was divided into five groups in parallel. Before inoculating the cells, the scaffolds were positioned in the 96-well plate. The scaffolds were tested for cytotoxicity with a CCK-8 kit (BestBio, Shanghai, China) on days 1, 3, 5, and 7, respectively. The absorbance values at 450 nm were measured using an enzyme marker.

##### Fluorescence Staining

The scaffolds were cut into 1 cm × 1.3 cm, soaked in ethanol for 2 h, washed 3 times with sterile PBS, and then exposed to UV light overnight. Experiments on cytotoxicity using MC3T3-E1 received the same treatment as the CCK-8 experiments. The medium was prepared using DMEM containing 10% FBS and 5% P/S. The scaffolds were placed into 24-well plates, and a 6 mm-diameter iron ring was placed on the top of each scaffold before the medium was added, with the intention of sinking the scaffolds to the bottom. Each well was inoculated with 1 × 10^4^ cells, 1 mL of DMEM medium was added, and live-dead staining was performed on the third day to observe the status of the cells on the scaffolds by using a fluorescent confocal microscope (FV3000, Olympus, Tokyo, Japan).

##### Validation of Antibacterial Activity

The antibacterial performance of the AgNPs/PLGA scaffolds was evaluated against *Staphylococcus aureus* (*S. aureus*, ATCC 25923) and *Escherichia coli* (*E. coli*, ATCC 25922). A single colony of each bacterium was placed in 15 mL centrifuge tubes containing 5 mL of Mueller Hinton Broth (MHB) medium and shaken overnight. At the end of the experiment, the concentration of bacteria was diluted to 10^5^ colony-forming units (CFUs)/mL [30]. After being printed, the scaffolds were dried in an oven at 37 °C. The scaffolds were then sliced into discs measuring 5 mm in diameter. Before conducting inhibition experiments, the scaffolds were soaked in 75% ethanol for 2 h, and then exposed to UV light overnight. Bacteria inhibition experiments were performed using 96-well plates. Subsequently, five parallel groups of 5 mm diameter scaffolds were set up in 96-well plates, 100 μL of final concentration bacterial solution was added, and after overnight (12 h) incubation, the absorbance values of the bacterial solution (600 nm wavelength) were measured via a microplate reader (SPARK, Tecan, Männedorf, Switzerland) to predict the growth status of the bacteria in order to determine the bacterial inhibition effect of the scaffolds. In addition, the bacterial solution of each group was diluted 10^5^-fold, and the antibacterial effect of the scaffolds was verified by the number of bacteria colonies.

### 2.5. Statistical Analysis

All data were analyzed using two-tailed, unpaired Student’s *t*-tests. In addition, the differences between groups were shown as follows: ns represent no significance; *^/#/&^ *p* < 0.05; **^/##/&&^ *p* < 0.01.

## 3. Results and Discussion

### 3.1. Properties of Raw AgNPs

It has been demonstrated that the particle size, concentration, and phenotype of AgNPs have a correlation with their antibacterial activity [31]. AgNPs have potent antibacterial effects due to their large surface area-to-volume ratio, which provides more contact sites for micro-organisms, allowing the nano-silver to maintain its antibacterial effect effectively [32]. The particle size of the AgNPs was approximately 100 nm, as measured by DLS (Supporting Information Figure S1A). The conductive adhesive was coated uniformly with AgNPs, and the AgNPs powder was observed by SEM at different magnifications, with a diameter of approximately 100 nm (Supporting Information Figure S1B).

### 3.2. The Physical and Morphological Analysis of the AgNPs/PLGA Scaffolds

We printed scaffolds with different concentrations of AgNPs, by mass percentage: 3AgNPs/PLGA (3% AgNPs), 1AgNPs/PLGA (1% AgNPs), and 0.1AgNPs/PLGA (0.1% AgNPs), as well as a control PLGA (0% AgNPs/PLGA). PLGA has been approved for human use by the US Food and Drug Administration (FDA) and the European Medicines Agency (EMA) as being biocompatible, degradable and safety, and has been used extensively in research into medical engineering materials and drug delivery systems [33,34,35]. Thus, PLGA with high biosafety was selected as a carrier for AgNPs in this study. Nanoparticles have a high surface energy due to their large specific surface area, which places them in a highly unstable energy state. As a result, nanoparticles have a tendency to agglomerate, which will lead to a large increase in local silver concentration and consequently affect cellular activity [36]. Therefore, the homogeneous distribution of the AgNPs is essential for the antimicrobial performance of the scaffolds. In this study, SEM demonstrated that AgNPs were distributed uniformly within the AgNPs/PLGA scaffolds (Supporting Information Figure S2A). The presence of AgNPs in AgNPs/PLGA scaffolds was confirmed by EDS (Supporting Information Figure S2B).

**Figure 1:**
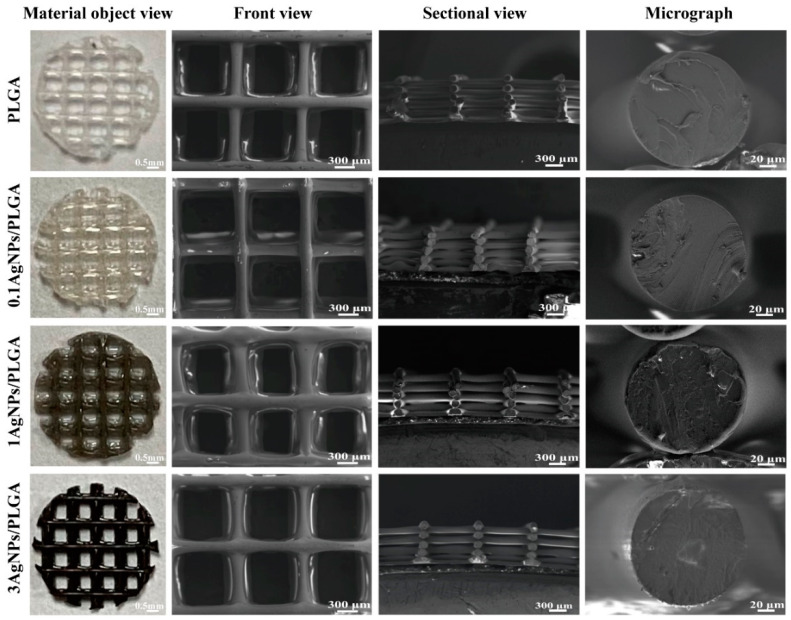
Material object view of different scaffolds and SEM images of scaffolds from different sides: front view, sectional view, and micrograph view.

The DIW-printed AgNPs/PLGA scaffolds have a uniform porous structure with interconnected pores, as seen in the three-dimensional representation in Figure 1. The porous microstructure is an important characteristic of the bone scaffold because its high specific surface area facilitates protein adsorption and provides anchoring sites for osteoblasts [37]. When the pore structure of the scaffold is greater than 300 μm, it facilitates the infiltration, adhesion, and migration of bone tissue cells, as well as the growth of blood vessels, which sees benefits in terms of providing nutrients for repairing bone tissue [20]; the minimum acceptable size of the scaffold is approximately 100 µm [38], as pore sizes smaller than 100 µm may prevent angiogenesis [39]. The pore size structure of our scaffolds is greater than 300 µm (Figure 1: front view). Additionally, the presence of gaps between each layer of the scaffolds suggests that the scaffolds were interconnected, and the thickness of the single strand is about 0.1mm (Figure 1. sectional view). It has been demonstrated in vivo and in vitro that pore interconnectivity has a positive effect on the rate of bone deposition and deep cell infiltration [40]. Microscopic images of the scaffolds (Figure 1) revealed uniform fiber diameters (0.15 mm) and a tight composition within the fibers, indicating that the 3D-printed scaffolds had structural homogeneity in terms of filament diameter as well. Figure 1 demonstrates that the printed scaffolds had high structural homogeneity and controlled geometry under the aforementioned process conditions, indicating that the printing parameters can be adjusted according to the bone tissue repair at various sites for therapeutic purposes.

### 3.3. The Mechanical Properties of the AgNPs/PLGA Scaffolds

**Figure 2:**
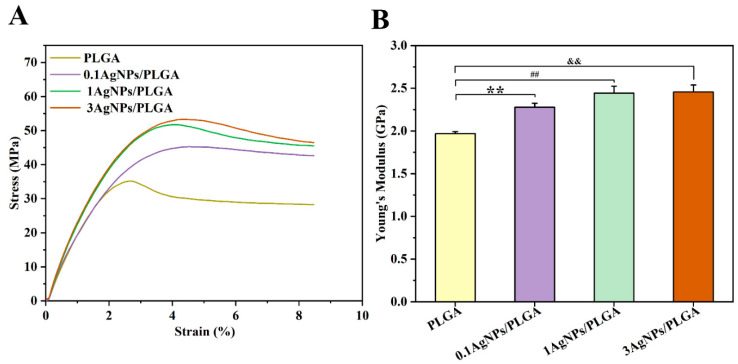
Mechanical properties of the 3D-printed scaffolds. (**A**) Stress-strain curves of the scaffolds; (**B**) the Young’s modulus of the scaffolds (**, ^##^, and ^&&^ represent *p* < 0.01).

The scaffolds used in bone tissue engineering are supposed to have mechanical properties that are compatible with the intended tissue. In the process of bone remodeling and repairing, mechanical force plays an important regulatory role [41]. Tensile testing is widely accepted as a basic test for testing the strength of materials. The most commonly used test is the uniaxial tensile test, in which a specimen is stretched uniformly at a specified rate, and the tensile force and elongation are recorded. The material’s parameters, such as stress-strain curves and Young’s modulus, are deduced from this [42]. Therefore, to characterize the scaffolds’ mechanical properties, we conducted tensile tests. The tests were given an average of three repetitions for each sample [43]. According to the stress-strain curves (Figure 2A), the tensile strength of PLGA was 34.9 ± 0.23 MPa. Meanwhile, with the addition of AgNPs, the tensile strength increased significantly. Specifically, the tensile strength of 0.1AgNPs/PLGA, which was 45 ± 0.17 MPa, and the 1AgNPs/PLGA and 3AgNPs/PLGA were 51.3 ± 0.67 MPa and 52.7 ± 0.51 MPa, respectively. Figure 2B displays that the Young’s modulus of PLGA was 1.96 ± 0.03 GPa. For the AgNPs/PLGA scaffolds, the Young’s modulus, respectively, increased to 2.28 ± 0.04 GPa, 2.44 ± 0.08 GPa, and 2.46 ± 0.08 GPa with increasing AgNP concentration. Fillers typically restrict the mobility of the polymer matrix and increase the rigidity of the scaffolds [44]. In this study, AgNPs were also utilized as fillers to increase the rigidity of the scaffolds because the addition of silver enhanced the Young’s modulus of the AgNPs/PLGA scaffolds, which is consistent with previous research [20]. However, the Young’s modulus difference between the 1AgNPs/PLGA and 3AgNPs/PLGA was not statistically significant (2.44 vs. 2.46, *p* = 0.535), indicating that the Young’s modulus of the scaffolds may not increase indefinitely with increasing silver concentration. Here, the Young’s modulus values of the scaffolds ranged between cancellous bone (0.02 GPa to 0.5 GPa) and cortical bone (3 GPa to 30 GPa) [45]; these results also support the utilization of AgNPs/PLGA scaffolds for bone tissue engineering applications.

### 3.4. The Release of Silver Ions from the AgNPs/PLGA Scaffolds

**Figure 3:**
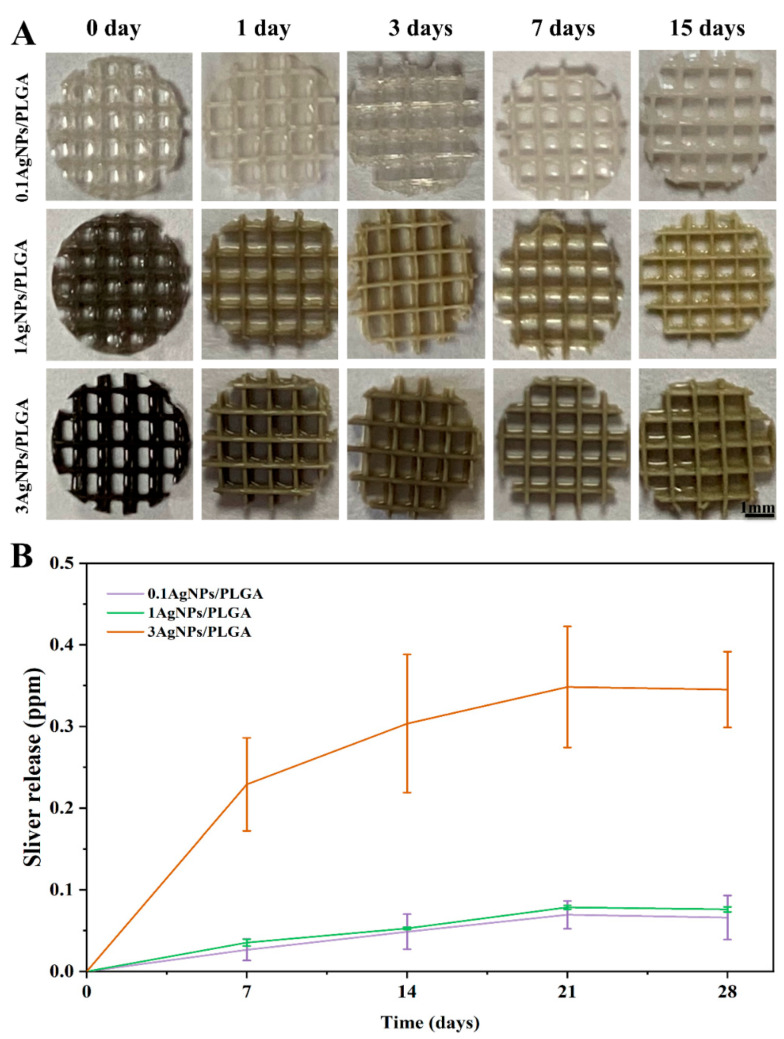
(**A**) Object view of the scaffolds with different contents of AgNPs: 0.1 wt%, 1 wt%, and 3 wt%, respectively, for 0, 1, 3, 7, and 15 days (every image follows the scale bar of the image at the end). (**B**) In vitro sliver release curves of scaffolds with different contents of AgNPs: 0.1 wt% (purple), 1 wt% (green), and 3 wt% (orange), respectively, for 7, 14, 21, and 28 days.

The release rate of silver ions from the AgNPs/PLGA scaffold materials was evaluated to identify the most suitable scaffold for repairing bone defects. It is important for the scaffolds to maintain a stable and sustained release of silver ions to preserve their bacteriostatic effect over time and provide effective antimicrobial performance [46]. The release rate must be maintained at a concentration of at least 0.1 ppm [47,48] in order to provide effective antimicrobial performance. The tests were given an average of three repetitions for each sample. As depicted in Figure 3B, the silver ions release rate was rapid on the seventh day, with 0.23 ppm for 3AgNPs/PLGA compared to 0.035 ppm and 0.026 ppm for 1AgNPs/PLGA and 0.1AgNPs/PLGA, respectively. After 7 days, the release rate slowed down but continued to increase. The release of silver ions reached a plateau on the 21st day. The continued release of silver ions on day 28 indicated that the silver ions were being released continuously over time, which is a requirement for maintaining long-term antimicrobial effects for scaffolds for bone tissue repair. This is consistent with previous reports [49]. After immersing the scaffolds in deionized water, we noticed that the color gradually lightened as the immersion time increased (Figure 3A), indicating that the silver ions were released from the scaffolds. The preceding findings laid the groundwork for us to validate the antibacterial effect in the following analysis.

### 3.5. Chemical Characterization of the Scaffolds

It is well known that chemical bonding information can be examined via FTIR [50]. Figure S3 displays the FTIR spectra of four scaffolds: PLGA, 0.1AgNPs/PLGA, 1AgNPs/PLGA, and 3AgNPs/PLGA. In the FTIR spectra of the PLGA copolymer, a strong peak at 1753 cm^−1^ was observed, which corresponds to the stretching vibration of etheric C=O bonds in the structure of PLGA polyester. Additionally, the absorption peaks at 1090 and 1182 cm^−1^ were assigned to etheric C-O bonds, while the peaks at 2885 and 2940 cm^−1^ indicated the aliphatic CH_2_ groups of the polymer backbone. These results were consistent with previous reports [51]. For all the AgNPs/PLGA scaffolds, including 0.1AgNPs/PLGA, 1AgNPs/PLGA, and 3AgNPs/PLGA, all the peaks that correspond to the PLGA section were present in the spectra. No new peak was observed in the AgNPs/PLGA scaffolds when compared to PLGA. Therefore, no new chemical bonding was formed between the AgNPs and PLGA.

### 3.6. Biological Characterization of the AgNPs/PLGA Scaffolds

**Figure 4:**
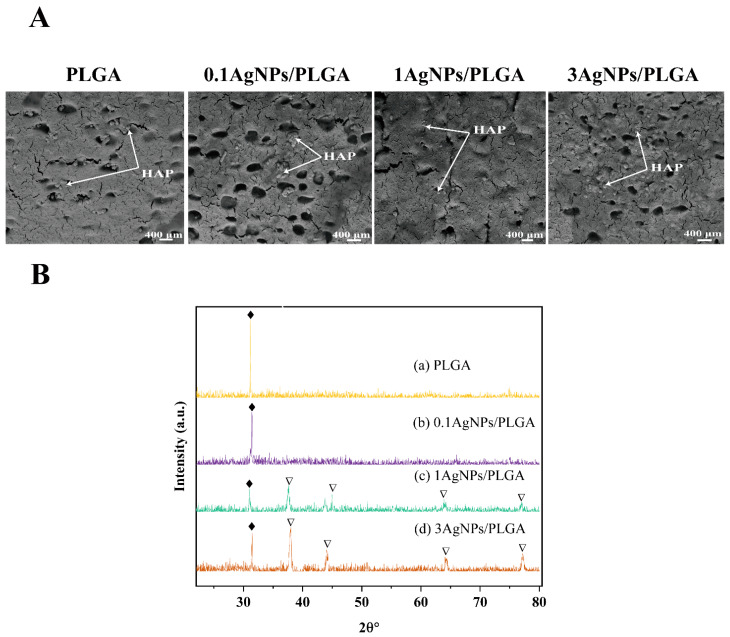
(**A**) SEM micrographs of the HAP grown on the surface of the 3D-printed scaffolds. (**B**) XRD spectra of the HAP grown on the surface of the scaffolds; the diamonds (♦) represent HAP and the inverted triangles (∇) represent AgNPs.

Biological characterization is another important measurement regarding bone implant materials. After conducting an in vitro bioactivity test (immersion in SBF), the surfaces were observed under SEM, and the micrographs obtained are shown in Figure 4A. Mineralized HAP growth on the surfaces is clearly visible on all the scaffolds. The growth of the HAP was further characterized by XRD, and the results are presented in Figure 4B. In the XRD images, the characteristic peak of HAP is present at 31.89° for each scaffold [9]. Additionally, four peaks were found in the 1AgNPs/PLGA scaffolds and 3AgNPs/PLGA scaffolds at 38°, 44°, 65°, and 77°, which represent the Bragg reflections of Ag (111), (200), (220), and (311) the reflection planes [52,53]. This also confirms that the scaffolds had mixed with the AgNPs. However, due to the low amount of AgNPs added to the 0.1 AgNPs/PLGA scaffolds, no characteristic peaks for AgNPs were observed.

**Figure 5:**
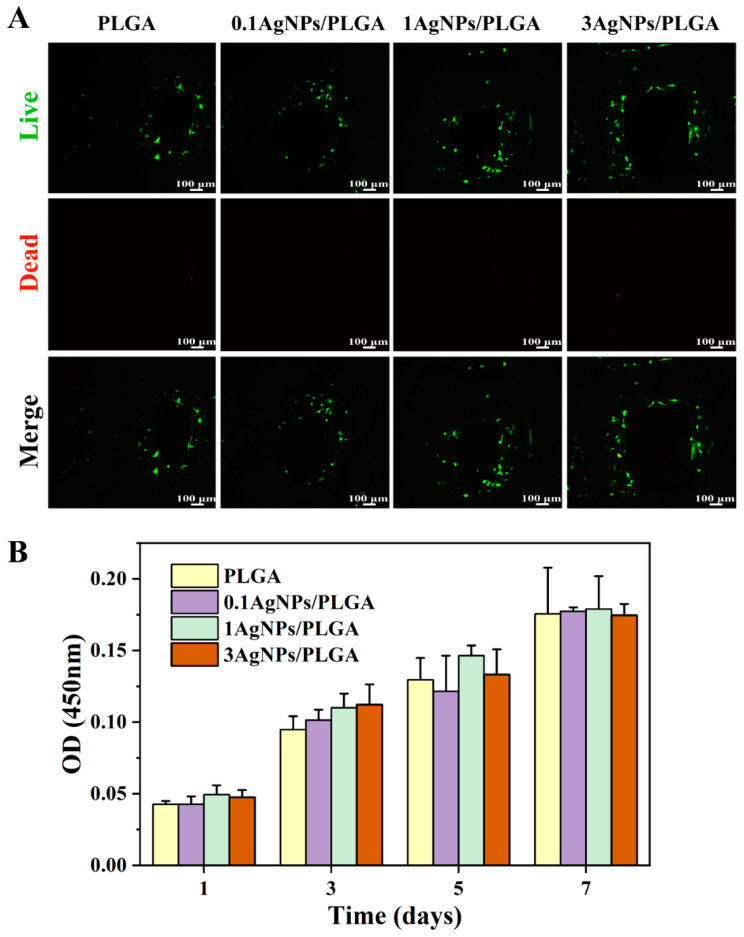
Qualitative analysis of the in vitro biological properties of the scaffolds. (**A**) Confocal laser images of MC3T3-E1 cells growing on the surface of the scaffolds for 3 days after live (green)/death (red) staining. (**B**) Absorbance values at 450 nm of the adhered MC3T3-E1 cells on different scaffolds after 1, 3, 5, and 7 days of incubation, as detected via CCK-8 assay.

In addition to mechanical properties, biocompatibility is an important property in bone implant materials. We evaluated the suitability of the AgNPs/PLGA scaffolds for the repair of human bone tissue by verifying their cytotoxicity. In this study, the cytocompatibility of the AgNPs/PLGA scaffolds was evaluated using MC3T3-E1, a suitable osteoblast cell line for in vitro testing. Previous studies have demonstrated that materials with an appropriate amount of silver loaded onto their surface exhibit good cellular compatibility and proliferation rates [54]. The viability of MC3T3-E1 cells was assayed using live/dead staining after 3 days of cell culture [55], where the live cells were stained with calcein acetoxymethyl ester (Calcein-AM: green), and the dead cells were stained with propidium iodide (PI: red) under a fluorescence microscope. In this experiment, it was hard to observe the dead cells at the highest AgNP concentration, indicating that there was almost no cytotoxicity and an effective bacterial inhibition effect (Figure 5A). Consistent with the live-dead staining results, the CCK-8 experiment revealed (Figure 5B) that increasing AgNP concentration had no effect on cell viability on days 1, 3, 5, and 7, and that neither the experimental nor control groups exhibited significant cytotoxicity. Some studies have shown that excessive amounts of AgNPs can be toxic to humans [56]. In our study, the scaffolds containing AgNPs were shown to be non-toxic via cellular experiments. As can be seen in Figure 3B, the release of silver from the scaffolds is continuous due to the advantages of the DIW printing technology, which avoided cytotoxicity due to the sudden release of the AgNPs. As a result, the scaffolds that we printed that were modified with AgNPs had good biocompatibility and can be used in humans for in vivo bone tissue repair.

### 3.7. Antibacterial Activity of the AgNPs/PLGA Scaffolds

**Figure 6:**
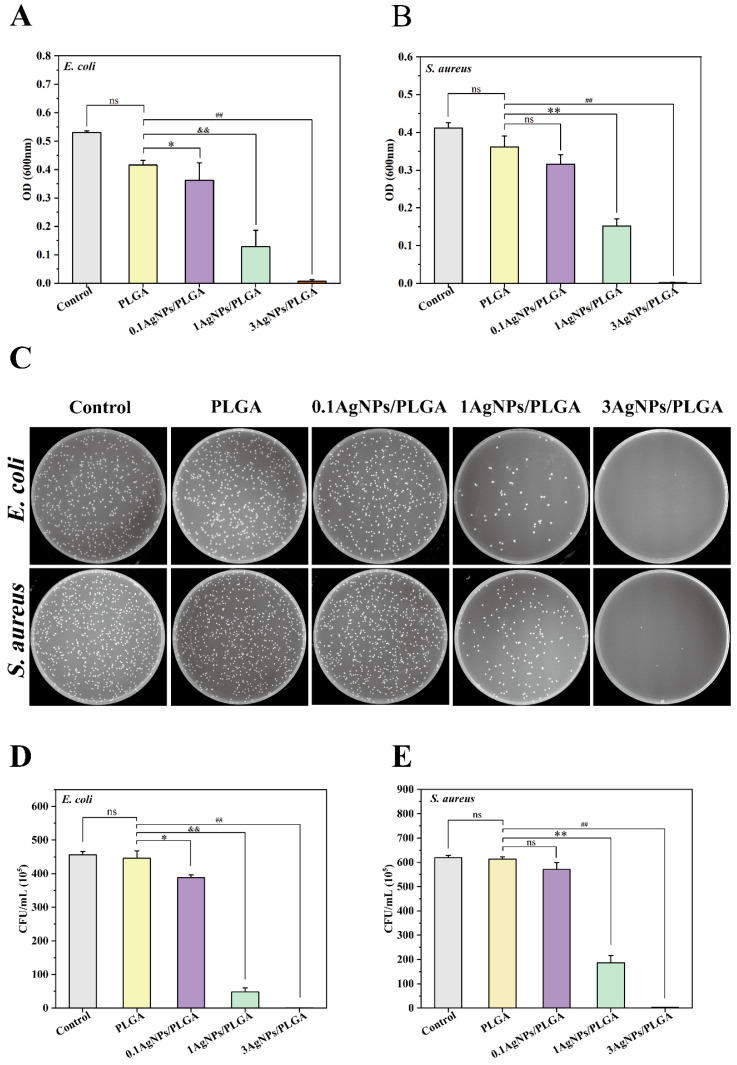
Analysis of viability of *E. coli* (**A**) and *S. aureus* (**B**) regarding the different AgNPs/PLGA scaffolds. (**C**) The antibacterial effect of different AgNPs/PLGA scaffolds was demonstrated via plate spreading. (**D**,**E**) The bacteria were quantified by counting CFU after treatment with the scaffolds. (ns represent no significance; * represent *p* < 0.05; **, ^##^, and ^&&^ represent *p* < 0.01).

It has been reported that a certain range of Ag concentration can kill bacteria without damaging the functions of mammalian cells [49]. AgNPs with a wide antibacterial spectrum have activity against both Gram-positive and Gram-negative bacteria and have not yet been observed to cause bacterial resistance to clinically relevant pathogens [54]. In our study, the decrease in OD600 absorbance indicated that the scaffolds could inhibit the growth of *E. coli* and *S. aureus* [9]. The obtained tests were given an average of three repetitions for each sample. The inhibition of the growth of *E. coli* became more pronounced with the increase in AgNP concentration. Additionally, the addition of AgNPs obviously inhibited the bacterial growth of *S. aureus* (Figure 6A,B). 1AgNPs/PLGA and 3AgNPs/PLGA exhibited statistically significant inhibition effects when compared to PLGA (*p* < 0.001), whereas 0.1AgNPs/PLGA exhibited no statistical difference when compared to PLGA (*p* = 0.107). When the concentration of AgNPs reached 3%, it essentially completely inhibited the growth of both *S. aureus* and *E. coli*, and, at this concentration, there was no cell toxicity (Figure 5). After incubation, followed by spreading the diluted bacteria on Mueller-Hinton agar plates, we observed that the 3AgNPs/PLGA plates had no sterile colony growth (Figure 6C). Inhibition increased in an AgNP proportion-dependent manner. According to the CFU counts [27], the plates with 0.1AgNPs/PLGA and 1AgNPs/PLGA scaffolds had fewer colonies than those with PLGA and the control groups. The 3AgNPs/PLGA scaffolds showed significant bacterial inhibition ability when compared to the control group scaffolds (*p* < 0.01), and there was no statistical difference between the PLGA and control groups in terms of colony count (*p* > 0.05). This bacterial inhibition efficiency result was consistent with that indicated by OD600 [9]; these findings further demonstrate the antibacterial effect of the AgNPs/PLGA scaffolds. The antibacterial effect was achieved via the release of silver ions from within the scaffolds. Hettinger et al. evaluated silver as a broad-spectrum bactericidal coating for biomedical implants [57]. In their study, the silver-containing layers on the implant surfaces impeded the growth of *S. aureus* and *E. coli* due to the release of silver ions from the coating layers. In addition, researchers have reported that graphene oxide silver nanoparticles (GO-AgNPs) exhibited excellent antibacterial activity against several Gram-negative and Gram-positive bacteria, and GO-AgNPs were capable of killing *P. aeruginosa* by releasing silver ions from finely dispersed AgNPs [58,59].

## 4. Conclusions

We successfully printed AgNPs/PLGA scaffolds using the DIW printing technique. This printing technique was able to homogeneously mix PLGA with AgNPs to achieve the slow release of AgNPs, which resulted in long-term bacterial inhibition. The SEM results showed that the scaffolds had a uniform porous structure with a pore size favorable to the infiltration and adhesion of bone tissue cells. Meanwhile, the mechanical properties of the scaffolds were enhanced after the addition of AgNPs to PLGA. The XRD results showed the formation of HAP on the scaffolds, which indicated the good bioactivity of the scaffolds, proving the presence of AgNPs on the scaffolds also. In addition, the in vitro cellular assay results showed that the composite scaffolds were biocompatible, non-cytotoxic, and could promote bone repair. In vitro, the scaffolds incorporating AgNPs showed good antibacterial effects against both *S. aureus* and *E. coli*. In order to verify the further application of the scaffolds, subsequent studies may consider in vivo animal experiments to further verify the antibacterial function of the scaffolds as well as their bone repair functionality.

## Data Availability

The data presented in this study are available upon reasonable request from the corresponding author.

## Supplementary Materials

The following supporting information can be downloaded at: https://www.mdpi.com/article/10.3390/ma16113895/s1, Figure S1: The particle size of the AgNPs powder. (A) DLS images and (B) SEM images; Figure S2: The distribution of the AgNPs in the scaffolds. (A) SEM images, (B) EDS images; Figure S3: The FTIR results of (a) PLGA, (b) 0.1AgNPs/PLGA, (c) 1AgNPs/PLGA and (d) 3AgNPs/PLGA.
