# Supplementary material for: Antibacterial 3D-Printed Silver Nanoparticle/Poly Lactic-Co-Glycolic Acid (PLGA) Scaffolds for Bone Tissue Engineering
Source: Materials (Basel). 2023 May 23;16(11):3895. doi: 10.3390/ma16113895 (PMC10253518; doi:10.3390/ma16113895)
Supplement: Supplementary file 1 [file materials-16-03895-s001.zip › materials-2258338-supplementary.pdf]

Supporting Information

Antibacterial 3D Printed Silver Nanoparticles/Poly Lac-tic-Co-Glycolic Acid (PLGA) Scaffolds for Bone Tissue Engineering

Fajun Chen[1,2], Jan Han[2,3], Chongjing Mu[4], Chuandi Yu[2,3], Zeyong Guo[2,3], Zhibo Ji[5], Lei Sun[5,6*], Yujuan Wang[2*], Junfeng Wang[1,2]

[1]Department of Anatomy, School of Basic Medicine, Anhui Medical University, No.81, Meishan Road, Shushan District, Hefei 230032, China

[2]High Magnetic Field Laboratory, Hefei Institutes of Physical Science, Chinese Academy of Sciences, Science Island, Hefei 230031, China

[3]Graduate School of University of Science and Technology of China, Hefei 230026, China

[4]The Affiliated Suzhou Hospital of Nanjing Medical University, 16 Baita West Road, Suzhou 215000, China

[5]Department of Stomatology, The Second Affiliated Hospital of Anhui Medical University, Hefei 230601, China

[6]Department of Oral Surgery, Ninth People's Hospital, College of Stomatology, Shanghai Jiao Tong University School of Medicine; Shanghai Key Laboratory of Stomatology & Shanghai Research Institute of Stomatology, National Clinical Research Center of Stomatology, Shanghai 200011, China

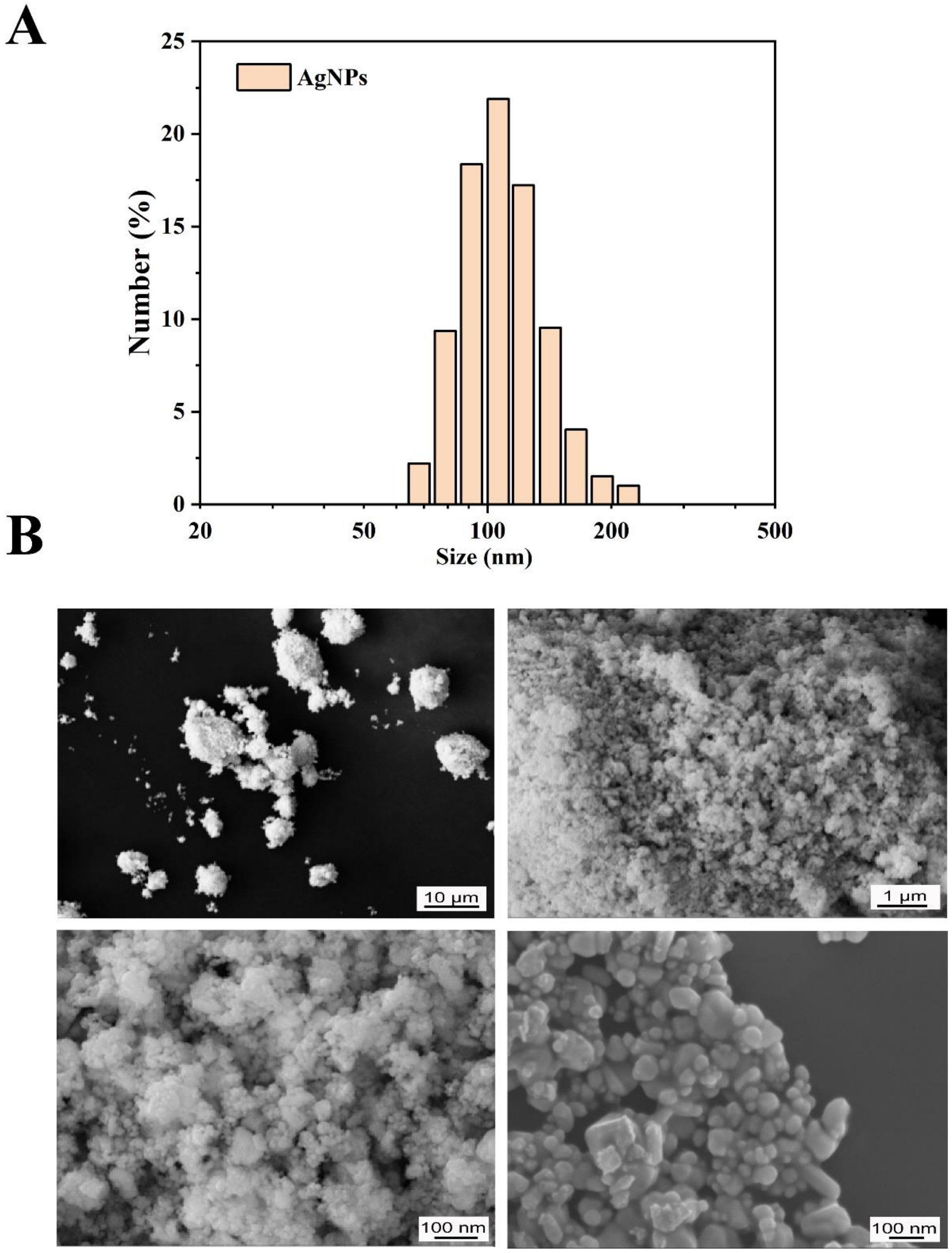

Figure S1. The particle size of the AgNPs powder. (A) DLS images and (B) SEM images.

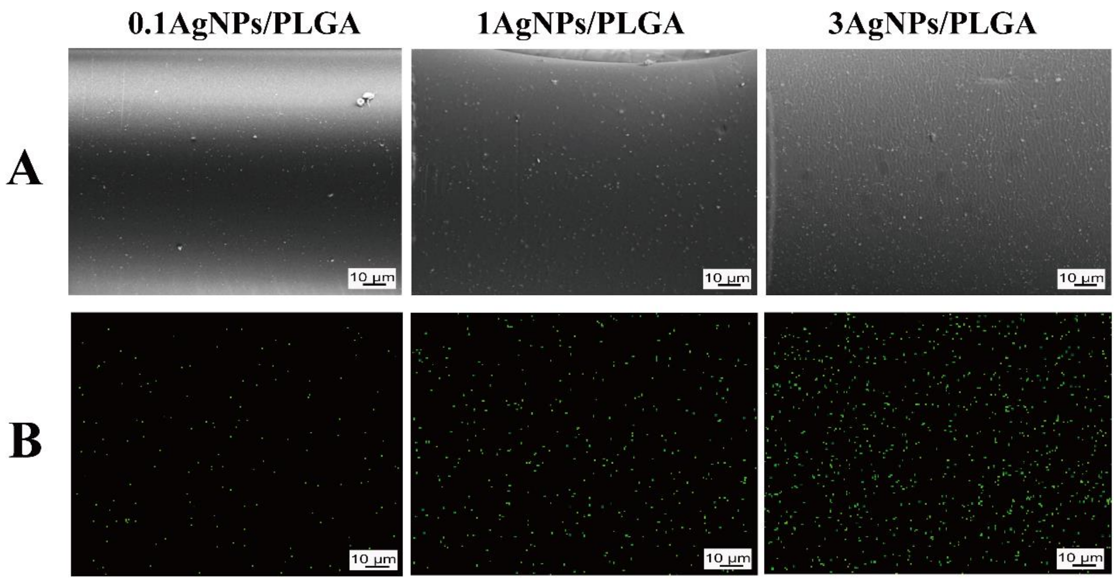

Figure S2. The distribution of the AgNPs in the scaffolds. (A) SEM images, (B) EDS images.

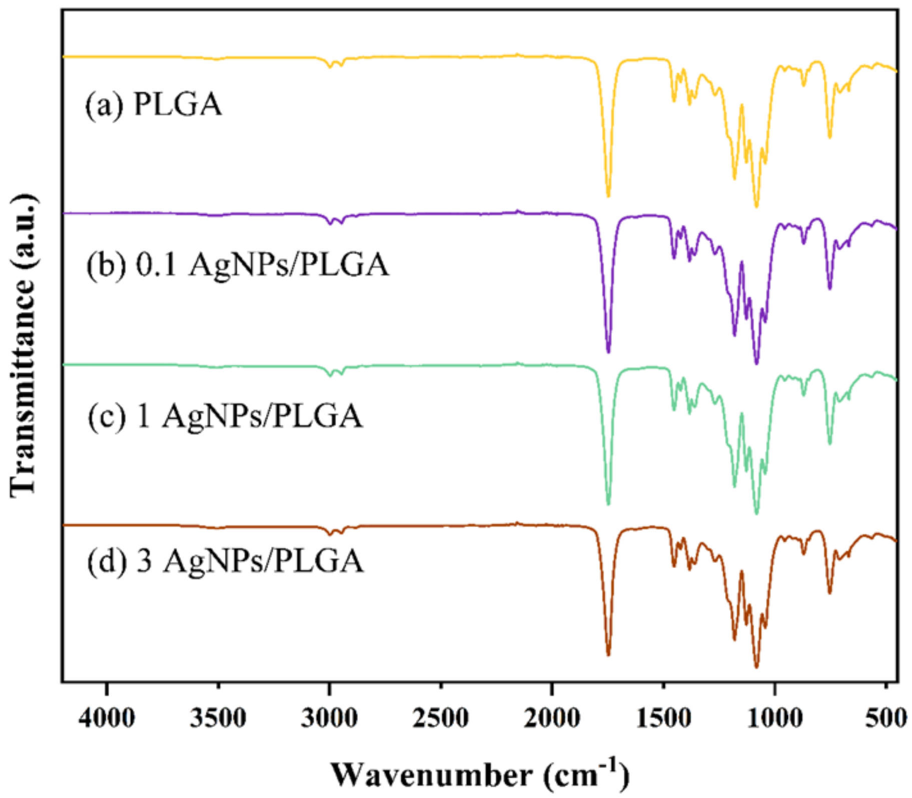

Figure S3. The FTIR results of (a) PLGA, (b) 0.1AgNPs/PLGA, (c) 1AgNPs/PLGA and (d) 3AgNPs/PLGA.
